# Galectin-9 Expression Predicts Favorable Clinical Outcome in Solid Tumors: A Systematic Review and Meta-Analysis

**DOI:** 10.3389/fphys.2018.00452

**Published:** 2018-04-26

**Authors:** Xiaoxiang Zhou, Lejia Sun, Dan Jing, Gang Xu, Jinmei Zhang, Li Lin, Jingjing Zhao, Zhuoran Yao, Hongfeng Lin

**Affiliations:** ^1^Department of Hepatobiliary Surgery, Weifang People’s Hospital, Weifang, China; ^2^Department of Liver Surgery, Peking Union Medical College Hospital, Peking Union Medical College and Chinese Academy of Medical Sciences, Beijing, China; ^3^Department of Obstetrics and Gynecology, Peking Union Medical College Hospital, Peking Union Medical College and Chinese Academy of Medical Sciences, Beijing, China

**Keywords:** galectin-9, favorable outcome, prognostic biomarker, solid tumor, metastasis, tumor invasion, meta-analysis

## Abstract

**Background and Objective:** Galectin-9 (Gal-9) is one of the galectin family members which are known as proteins with β-galactoside-binding affinity. Accumulative evidence suggest that Gal-9 plays multifaceted roles in tumor biology. However, the prognostic significance of Gal-9 in solid cancer patients remains controversial. The objective of the study was to clarify the prognostic significance of Gal-9 in solid tumors via meta-analysis.

**Methods:** We searched PubMed, Embase and the Cochrane library for studies that report the correlation between Gal-9 expression and prognosis or clinicopathological parameters in solid cancer patients from inception to October 2017, with no language restriction. We calculated pooled hazard ratio (HR) and 95% confidence interval (CI) to investigate the prognostic significance of Gal-9 expression in solid tumors. We also calculated Odds ratio (OR) to explore the association between Gal-9 expression and clinicopathological features.

**Results:** We included Fourteen studies with 2326 patients in our meta-analysis. The synthetic results revealed that high Gal-9 expression indicated improved overall survival (OS; HR = 0.70, 95% CI = 0.51–0.71, *P* = 0.006) but had no correlation with disease-free survival (DFS)/recurrence-free survival (RFS) (HR = 0.85, 95% CI = 0.51–1.41, *P* = 0.527) in solid tumors. In stratified analyses, high Gal-9 expression was significantly correlated with improved OS in hepatocellular carcinoma and colon cancer and with improved DFS/RFS in gastric cancer and non-small cell lung cancer. In addition, ethnicity and the method of data extraction didn’t affect the positive prognostic values of high Gal-9 expression. Moreover, high Gal-9 expression was significantly correlated with a smaller depth of invasion (TI/TII vs. TIII/TIV, OR = 2.80, 95% CI = 1.97–3.96, *P* < 0.001), an earlier histopathological stage (I/II vs. III/IV, OR = 3.00, 95% CI = 2.04–4.42, *P* < 0.001), negative lymph node metastasis (Presence vs. Absence, OR = 0.47, 95% CI = 0.25–0.89, *P* = 0.020) and negative distal tumor metastasis (Presence vs. Absence, OR = 13.85, 95% CI = 3.50–54.76, *P* < 0.001).

**Conclusion:** Gal-9 expression indicates beneficial outcome in patients with solid tumors and is correlated with the pathogenesis of solid tumors. Gal-9 may serve as a prognostic biomarker and an emerging therapeutic target against solid tumors.

## Introduction

Cancer is one of the leading causes of death worldwide. Surgery, radiation and chemotherapy are the commonly recommended first-line therapies for most cancer cases. Nevertheless, these treatments are not beneficial to all patients, and this lack of benefit may be due to tumor heterogeneity (Weiskirchen, 2016). Moreover, tumor recurrence following these treatments is common and has a predominantly negative impact on outcome, and existing methods cannot precisely predict the outcome for cancer patients. Therefore, novel prognostic markers and new therapeutic strategies are urgently needed. The hallmarks of cancer include cell death resistance, invasion and metastasis, and immune escape, among others (Hanahan and Weinberg, 2011). Thus, explorations aimed at preventing metastasis, inducing tumor cell death and strengthening antitumor immunity are key to future therapy.

Galectins are proteins harboring the affinity to bind β-galactoside and possess carbohydrate recognition domains (Barondes et al., 1994a,b). So far, investigators have identified 15 members in the galectin family, and galectin-9 (Gal-9) is one of those members (Gray et al., 2004). Three splice variants of Gal-9 are frequently reported in the publications: full-length Gal-9 (Gal-9FL), Gal-9 with a deletion of exon 5 (Gal-9-delta-5), and Gal-9 with deletions of exons 5 and 6 (Gal-9-delta-5/6) (Chabot et al., 2002). Accumulative evidence suggests that Gal-9 plays a multifaceted role in preventing cancer progression. As reported in breast cancer cells, cytoplasmic Gal-9 induces cancer cell aggregation (stabilizing cell-cell adhesion junctions) and inhibits cell invasion, detachment from the tumor, and attachment to the vascular endothelium (Irie et al., 2005; Yamauchi et al., 2006). Additional antitumor roles for Gal-9 have been reported in a recent *in vivo* study, in which Gal-9 induced apoptosis and inhibited the growth of hepatocellular carcinoma (HCC) cells (Fujita et al., 2015). Moreover, Gal-9 enhances the cytolytic activity against tumor of NK cells through expanding plasmacytoid cell-like macrophages in a melanoma murine model (Nobumoto et al., 2009). Paradoxically, Gal-9 is also involved in immune escape (Chou et al., 2018). Initial evidence suggested that Gal-9 was a ligand of Time-3 that interacted with Tim-3 and negatively regulated Th1 immunity (Zhu et al., 2005). A subsequent study demonstrated that CD8+ cytotoxic T cells could be induced apoptosis by Gal-9 (Wang et al., 2007). Gal-9 also facilitates the suppressive activity of regulatory T cells via activating DR3 signaling, which are well known to promote tumor immune invasion (Madireddi and Eun, 2017). Consistent with its immunosuppressive function, Gal-9 contributes to immune dysfunction in human HCC through the Tim-3/Gal-9 interaction (Li et al., 2012). Recently, the Tim-3-Gal-9 secretory pathway has been proposed as the mechanism underlies immune escape of human acute myeloid leukemia cells (Goncalves Silva et al., 2017). The divergent effects of Gal-9 that are involved in tumor immunity make the role of Gal-9 in tumor progression ambiguous.

Recently, increasing attention has been given to the prognostic value of Gal-9 in cancer patients. However, whether Gal-9 has prognostic value in patients with solid tumors remains unclear. Some evidence indicates that high expression of Gal-9 contributes to a better outcome for various solid tumors (Yamauchi et al., 2006; Holtan et al., 2012; Zhang et al., 2012; Gu et al., 2013; Wang et al., 2016; Liu et al., 2017; Sideras et al., 2017). Nevertheless, several studies have obtained inconclusive results or even opposing results, which may be due to the heterogeneity of different tumors with various origins, the divergent role of Gal-9 in tumor immunity, the diverse expression profiles of receptors, variability among study designs and the sizes of the samples. Fu et al reported that positive Gal-9 expression predicted a worse clinical outcome in patients with urinary tumors (Fu et al., 2015). Moreover, seven research groups reported that Gal-9 indicated a trend toward a poor clinical outcome or had no correlation with the prognosis of various cancers (Jiang et al., 2013; Kong et al., 2014; Schulkens et al., 2014; Grosset et al., 2016; Ohue et al., 2016; Choi et al., 2017; Melief et al., 2017).

Hence, a systematic analysis of the correlation between Gal-9 expression and the prognosis of solid cancer patients by means of a meta-analysis of existing available data is necessary. Herein, we assessed the correlation between Gal-9 expression and survival by pooling data from published publications. Consequently, we found that Gal-9 was a positive indicator in solid cancer patients. To the best of our knowledge, we report the first meta-analysis to clarify the prognostic implication of Gal-9 expression among solid cancer patients.

## Materials and Methods

This systematic review and meta-analysis was implemented following the Preferred Reporting Items for Systematic reviews and Meta-Analyses (PRISMA) guidelines (Moher et al., 2009).

### Literature Search Strategy

We systematically searched PubMed, Embase and the Cochrane library for literatures published up to October 2017, without restrictions on language or region. The following keywords were used to carry out the search: (Galectin-9 OR LGALS9 protein) AND (cancer OR neoplasm OR malignancy OR carcinoma OR tumor). We also referred to the reference and citation of the retrieved publications.

### Eligible Criteria

The following inclusion criteria were used in the meta-analysis: (1) the studies published as original articles; (2) the papers that investigated the correlation between Gal-9 expression and prognosis of solid cancer patients, such as overall survival (OS), disease-free survival (DFS), and recurrence-free survival (RFS) or clinicopathological features; (3) As to prognostic data, there were enough available data for directly extraction or indirectly estimate for hazard ratios (HR) and 95% confidence interval (CI); (3) the sample size of every study is more than fifty individuals; Studies were excluded if they failed to meet all criteria. For multiple publications reporting the same study or based on identical datasets, we used the most informative or most recent publication to avoid duplication. To ensure the reliability of the search results, two authors (XZ and LS) independently carried out the retrieval and screening in accordance with the standardized approach, and the consensus was reached by discussion.

### Data Collection and Quality Assessment

Two authors (XZ and LS) independently reviewed all included studies and extracted the data with a standardized protocol. If the authors couldn’t reach a consensus, a third researcher made the final decision. Following are the extracted context: the clinicopathological characteristic; first author’s name; publication year; ethnicity of the patients; number of patients; cancer type; trial design; median age; cutoff value of high Gal-9 expression; median follow-up time and the range; use of multivariate or univariate model; outcome; method used to detect Gal-9 expression; specimen type and localization of Gal-9. If there was no available HR and 95%CI for direct extraction, we extract the survival data from Kaplan–Meier curves by the software Engauge Digitizer version 4.1^1^ (Tierney et al., 2007). If both univariate and multivariate HR with 95% CI were available in one study, we chose the multivariate data to avoid confounders.

Also, two authors (XZ and LS) independently evaluated the quality of the included studies according to the Newcastle-Ottawa scale (NOS) criteria, a widely used tool for the quality assessment of observational or non-randomized studies (Wells et al., 2009). Each study was graded on a scale of zero to nine according to the selection, comparability and outcomes of the study cohorts. Final consensus for the NOS scores of every item was reached.

### Statistical Analyses

As the outcomes, DFS and RFS were regarded as having a similar meaning and therefore used as a single united parameter. We utilized Stata 12.0 software to perform the data analyses. OS and DFS/RFS were used to evaluate the prognostic effect of Gal-9 in solid tumors. Odds ratio (OR) was used to evaluate the correlation between Gal-9 expression and clinicopathological parameters. We used χ^2^ test and *I*^2^ statistic to evaluate the heterogeneity across studies (Higgins and Thompson, 2002). If *P* < 0.10 for the χ^2^ test or *I*^2^ > 50%, significant heterogeneity was considered to exist and the random effects model was applied (DerSimonian and Kacker, 2007). If not, a fixed-effects model was applied (Mantel and Haenszel, 1959). We performed cumulative meta-analysis by publication years to explore the trends of the results. We also performed the sensitivity analysis, in which one research was deleted every time to judge the impact of one research on the results. We used funnel plot, Begg’s test and Egger’s tests to investigate the publication bias quantitatively (Begg and Mazumdar, 1994; Egger et al., 1997). For all analyses, two-sided *P* < 0.05 was considered statistical significant.

## Results

### Study Selection

**FIGURE 1:**
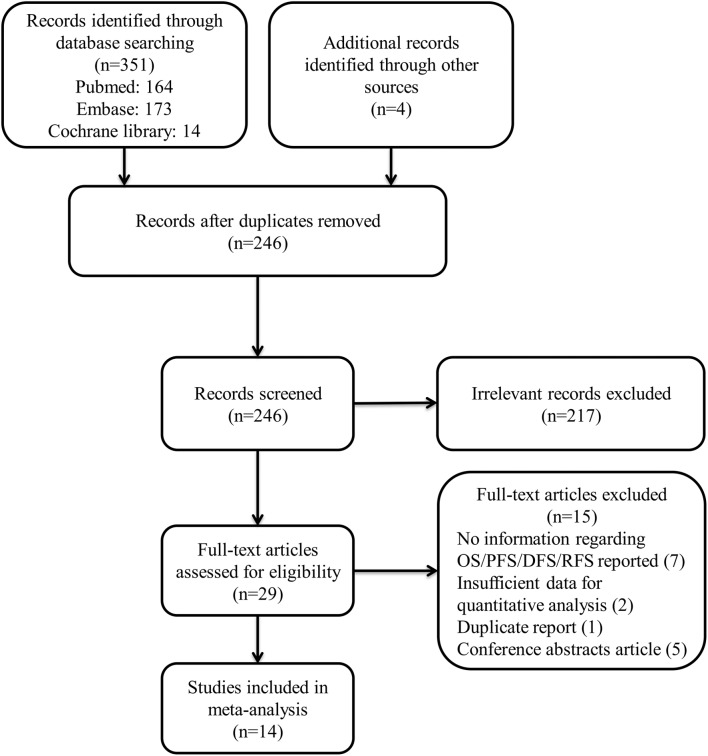
Flow diagram of the study selection process.

We identified 351 studies by systematic literature search. After reviewing the title and abstract, 215 were excluded for the reason that they were irrelevant to the topic. Finally, we included 14 from the remaining 29 studies. (Irie et al., 2005; Zhang et al., 2012; Gu et al., 2013; Jiang et al., 2013; Kong et al., 2014; Schulkens et al., 2014; Fu et al., 2015; Grosset et al., 2016; Ohue et al., 2016; Wang et al., 2016; Choi et al., 2017; Liu et al., 2017; Melief et al., 2017; Sideras et al., 2017). Following are the reasons why we excluded these studies: 7 had no information about OS/DFS/RFS or clinicopathological data reported; 2 lacked sufficient data for quantitative analysis; 5 were conference abstracts; and 1 was a duplicate report. The details of how we searched and selected the relevant studies are presented in **Figure 1**.

### Characteristics of the Included Studies

We included data from 2326 patients in this meta-analysis. The main features of the 14 studies were summed up in **Table 1**. These studies were published between 2005 and 2017. Twelve of the included studies reported a correlation between OS and Gal-9 expression, and six of the included studies further reported a relationship between DFS/RFS and Gal-9 expression. In addition, nine studies presented a correlation between clinicopathological features and Gal-9. These studies were carried out in 5 countries (10 studies were Eastern Asian cohorts, and 4 were Caucasian cohorts). Eight studies were prospective cohort trials, and six were retrospective cohort trials. Patients in all studies were reported to received resections. Regarding cancer type, studies related to HCC (*n* = 4) constituted the largest proportion among all included publications, followed by breast cancer (*n* = 2), gastric cancer (*n* = 2), non-small cell lung cancer (NSCLC) (*n* = 2), urinary tumors (*n* = 2), colon cancer (*n* = 1) and melanoma (*n* = 1). Most studies reported a localization of Gal-9 in cytoplasm, together with several studies reported an additional localization in nucleus or cell membrane or both. High Gal-9 expression levels across studies was defined by heterogeneous cutoff values, and the most common strategy was to regard the median score as the cutoff point. All studies comprised a median follow-up time ranging from 24.4 to 118 months. The most commonly used test method for Gal-9 was immunohistochemistry (IHC), with one study using qRT-PCR and another study using immunofluorescence staining (IFS). The collected HR were calculated with a multivariate model in nine studies and with a univariate model in five studies. Ten studies detected the Gal-9 expression with a Paraffin embedded block, one study utilized frozen tissue and four studies didn’t report their specimens specifically. The scores of all included studies were above five according to Newcastle-Ottawa scale (NOS), indicating that all studies were of high methodological quality (**Table 2**).

**Table 1:** Main characteristics of the eligible studies.

Author	Year	Ethnicity	Number of patient	Trial design	Cancer type	Cutoff value of positive Gal-9	Localization of Gal-9	Follow-up (months) Medium (range)	Outcomes	Model	Method	Specimen	NOS score
Irie	2005	Eastern Asian	84	PC	Breast cancer	*H*-score > 80	C	188	DFS	Uni	IHC	PEB	8
Zhang	2012	Eastern Asian	200	RC	HCC	Score > 2, (0–7)	C	≥60	OS	Multi	IHC	PEB	7
Gu	2013	Eastern Asian	147	PC	HCC	NA	C, M, N	24.4(9–43)	OS, DFS	Multi	IHC	NA	8
Jiang	2013	Eastern Asian	183	PC	Gastric cancer	*H*-score > 200	C	40(3–135)	OS	Uni	IHC	PEB	8
Kong	2014	Eastern Asian	197	PC	HCC	*H*-score > 100	C	21(12–56)	OS	Uni	IHC	PEB	8
Schulkens	2014	Caucasian	87	RC	NSCLC	≥median	NA	≥60	OS, DFS	Uni	qRT-PCR	Frozen tissue	5
Fu	2015	Eastern Asian	196	RC	Urinary tumors	*H*-score ≥ median	NA	106(12–120)	OS, RFS	Multi	IHC	PEB	7
Grosset	2016	Caucasian	98	RC	Breast cancer	NA	C, N	60	DFS	Uni	IHC	PEB	5
Ohue	2016	Eastern Asian	120	PC	NSCLC	Score = =3,4,6	C, M	NA	OS	Multi	IHC	PEB	8
Wang	2016	Eastern Asian	90	PC	Colon cancer	Score = =2,3,4	C	96	OS	Multi	IHC	PEB	8
Choi	2017	Eastern Asian	619	PC	Gastric cancer	≥10% of cells	HA	65.7(0–79)	OS	Multi	IHC	PEB	8
Liu	2017	Eastern Asian	202	RC	Urinary tumors	*H*-score = 58–157	C	60.5	OS, RFS	Multi	IHC	PEB	7
Melief	2017	Caucasian	73	PC	Melanoma	>Median *Z*-cores	NA	Upto 120	OS	Multi	IFS	PEB	8
Sideras	2017	Caucasian	94	RC	HCC	Score = 1,2,3 (0–3)	C	NA	OS	Multi	IHC	PEB	7


*PC, prospective cohort; RC, retrospective cohort; HCC: hepatocellular carcinoma; NSCLC, non-small cell lung cancer; C, cytoplasm; M, cell membrane; N, nucleus; OS, overall survival; DFS, disease-free survival; RFS, recurrence-free survival; Multi, multivariate; Uni, univariate; IHC, immunohistochemistry; IFS, immunofluorescence staining; qRT-PCR, quantitative real-time polymerase chain reaction; PEB, paraffin embedded block; N.A., not available.*

**Table 2:** The Newcastle-Ottawa Scale (NOS) quality assessment of the enrolled studies.

Study ID		Selection		Comparability			Outcome		Total
				
	Representativeness of the exposed cohort	Selection of the non-exposed cohort	Ascertainment of exposure	Demonstration that outcome of interest was not present at start of study	Comparability of cohorts on the basis of the design or analysis (study adjusts for age^∗^, ex^∗^)	Assessment of outcome	Was follow-up long enough for outcomes to occur	Adequacy of follow up of cohorts	
Irie et al., 2005	-	^∗^	^∗^	^∗^	^∗∗^	^∗^	^∗^	^∗^	8
Zhang et al., 2012	-	^∗^	^∗^	-	^∗∗^	^∗^	^∗^	^∗^	7
Gu et al., 2013	-	^∗^	^∗^	^∗^	^∗∗^	^∗^	^∗^	^∗^	8
Jiang et al., 2013	-	^∗^	^∗^	^∗^	^∗∗^	^∗^	^∗^	^∗^	8
Kong et al., 2014	-	^∗^	^∗^	^∗^	^∗∗^	^∗^	^∗^	^∗^	8
Schulkens et al., 2014	-	^∗^	^∗^	-	-	^∗^	^∗^	^∗^	5
Fu et al., 2015	-	^∗^	^∗^	-	^∗∗^	^∗^	^∗^	^∗^	7
Grosset et al., 2016	-	^∗^	^∗^	-	-	^∗^	^∗^	^∗^	5
Ohue et al., 2016	-	^∗^	^∗^	^∗^	^∗∗^	^∗^	^∗^	^∗^	8
Wang et al., 2016	-	^∗^	^∗^	^∗^	^∗∗^	^∗^	^∗^	^∗^	8
Choi et al., 2017	-	^∗^	^∗^	^∗^	^∗∗^	^∗^	^∗^	^∗^	8
Liu et al., 2017	-	^∗^	^∗^	-	^∗∗^	^∗^	^∗^	^∗^	7
Melief et al., 2017	-	^∗^	^∗^	^∗^	^∗∗^	^∗^	^∗^	^∗^	8
Sideras et al., 2017	-	^∗^	^∗^	-	^∗∗^	^∗^	^∗^	^∗^	7


*^∗^Means one score, ^∗∗^means two scores.*

### Correlation Between Gal-9 Expression and OS

**FIGURE 2:**
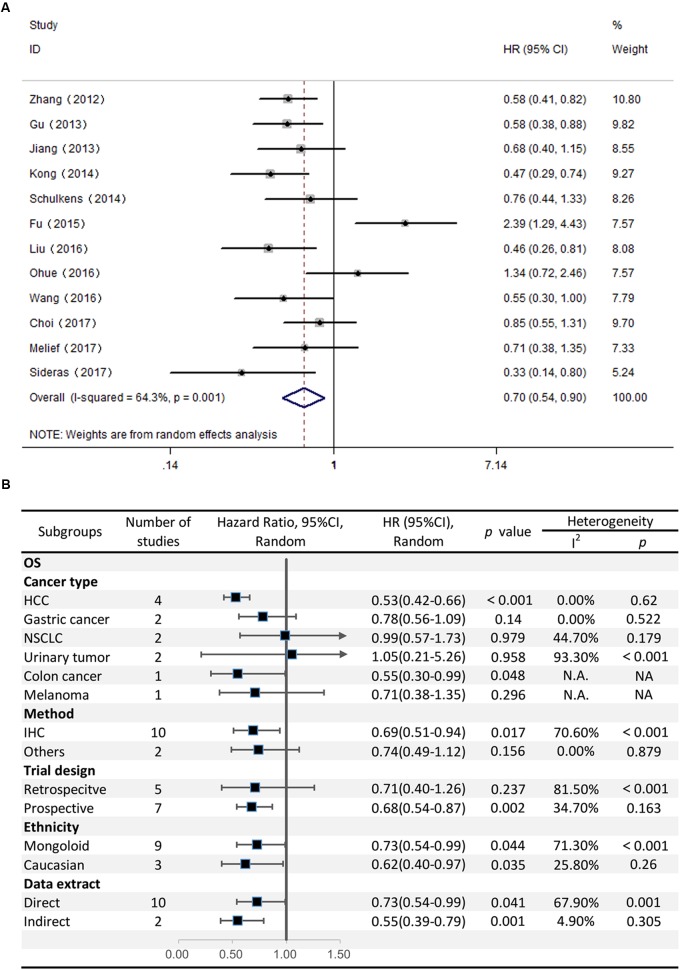
**(A)** Meta-analysis of the correlation between Gal-9 expression and OS among patients with solid tumors. **(B)** Subgroup analyses of the association between Gal-9 and OS.

Twelve studies comprising 2208 patients reported OS. This meta-analysis revealed that high Gal-9 expression was associated with improved OS among patients with various solid tumors (HR = 0.70, 95% CI = 0.51–0.71, *P* = 0.006) (**Figure 2A**). This pooled meta-analysis was carried out by the random effects model on account of significant heterogeneity (*I*^2^ = 64.3%, *P* = 0.001). To further explore the potential sources of heterogeneity, we utilized subgroup analyses, which is summarized in **Figure 2B**.

Subgroup analyses regarding cancer type clarified that increased Gal-9 expression was significantly correlated with longer OS for patients with HCC (pooled HR = 0.53, 95% CI = 0.42–0.66), along with no heterogeneity (*I*^2^ = 0.00%, *P* = 0.62). Nevertheless, no significant association was evident between Gal-9 expression and OS among patients with gastric cancer (pooled HR = 0.78, 95% CI = 0.56–1.09, *P* = 0.140), NSCLC (pooled HR = 0.99, 95% CI = 0.57–1.73, *P* = 0.979), urinary tumors (pooled HR = 1.04, 95% CI = 0.21–5.26, *P* = 0.958). By the way, we couldn’t get a pooled HR from colon cancer or melanoma because there’s only a single study reporting the prognostic data for OS.

**FIGURE 3:**
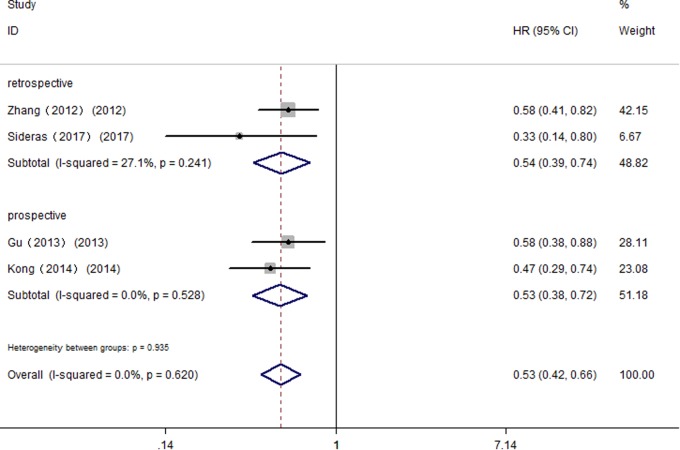
Subgroup analyses by trial design of the association between Gal-9 and OS in patients with HCC.

Further, we performed subgroup analyses by ethnicity. The results revealed that Gal-9 predicted improved OS in Eastern Asian populations (pooled HR = 0.73, 95% CI = 0.54–0.99) and in Caucasian populations (pooled HR = 0.62, 95% CI = 0.40–0.97). We also performed subgroup analyses regarding the data extraction method, for the reason that the HRs and 95% *Cis* in some included studies were indirectly estimated (as described in section “Materials and Methods”) and might be less reliable. The results showed that high Gal-9 expression was associated with improved OS in studies extracting data directly (pooled HR = 0.73, 95% CI = 0.54–0.99), which was in line with the data extracting indirectly (pooled HR = 0.55, 95% CI = 0.39–0.80). Nevertheless, the pooled results were not significant in studies applying other detection method (pooled HR = 0.74, 95% CI = 0.49–1.12, *P* = 0.156). Considering that IHC was the most widely used method in clinical practice, our results still made sense (IHC: pooled HR = 0.69, 95% CI = 0.51–0.94). In addition, pooled results were not significant in studies applying retrospective trial design (pooled HR = 0.71, 95% CI = 0.40–1.26, *P* = 0.237), with significant heterogeneity (*I*^2^ = 81.50%, *P* < 0.001). To further explore the difference between trial design in homogeneous population. We performed subgroup analyses by trial design in HCC with fixed-effects model. The results displayed that pooled HR in two subgroups were both significant (prospective: pooled HR = 0.53, 95% CI = 0.38–0.72, *P* < 0.001; retrospective: pooled HR = 0.54, 95% CI = 0.39–0.74, *P* < 0.001) (**Figure 3**), and no significant heterogeneity was observed (prospective: *I*^2^ = 0.00%, *P* = 0.528; retrospective: *I*^2^ = 27.10%, *P* = 0.241).

### Correlation Between Gal-9 Expression and DFS/RFS

**FIGURE 4:**
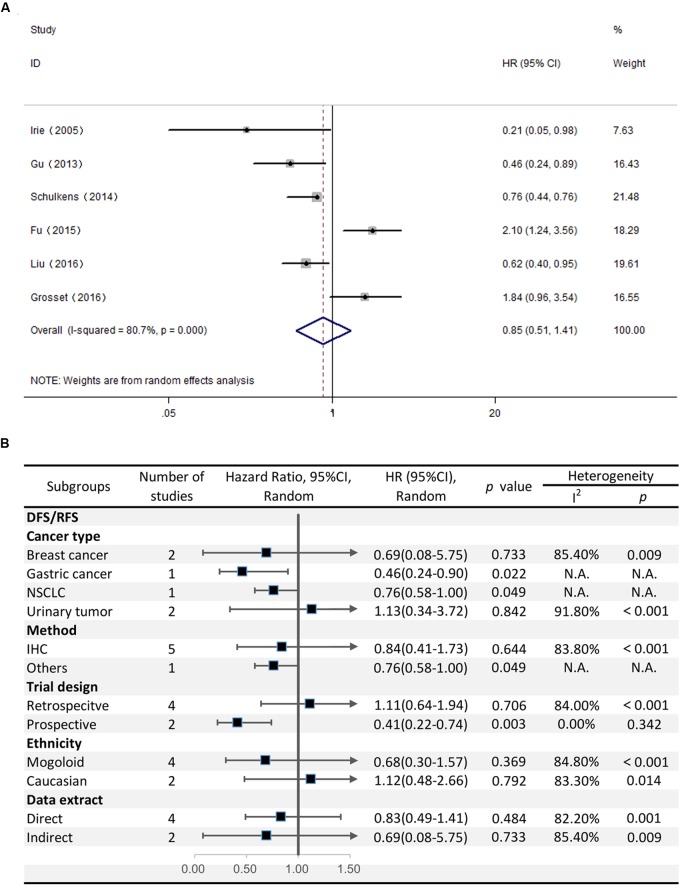
**(A)** Meta-analysis of the correlation between Gal-9 expression and DFS/RFS among patients with solid tumors. **(B)** Subgroup analyses of the association between Gal-9 and DFS/RFS.

The HRs for DFS/RFS were available in six studies comprising a total of 814 patients. Unlike OS, Gal-9 expression had no significant correlation with DFS/RFS (HR = 0.85, 95% CI = 0.51–1.41, *P* = 0.527) (**Figure 4A**). Because significant heterogeneity was observed (*I*^2^ = 80.7%, *P* < 0.001), a random effects model was applied to determine the summary of DFS/RFS.

With respect to the stratified analysis by cancer type, no significant association was evident between Gal-9 expression and DFS/RFS for breast cancer (pooled HR = 0.69, 95% CI = 0.08–5.75, *P* = 0.733) or urinary tumors (pooled HR = 1.13, 95% CI = 0.34–3.72, *P* = 0.84). We couldn’t get a pooled HR from gastric cancer or NSCLC because there’s only a single study reporting the prognostic data for OS.

Subgroup analyses revealed that the method of detection (IHC: pooled HR = 0.844, 95% CI = 0.41–1.73, *P* = 0.644; others: pooled HR = 0.76, 95% CI = 0.58–1.00, *P* = 0.049) and trial design (prospective: pooled HR = 0.41, 95% CI = 0.22–0.74, *P* = 0.003; retrospective: pooled HR = 1.113, 95% CI = 0.64–1.94, *P* = 0.706) had different effects on DFS/RFS. Moreover, no evident correlation was observed between Gal-9 expression level and DFS/RFS in any subgroup divided by ethnicity and the method of data extraction (**Figure 4B**).

### Implications of Cumulative Meta-Analysis

**FIGURE 5:**
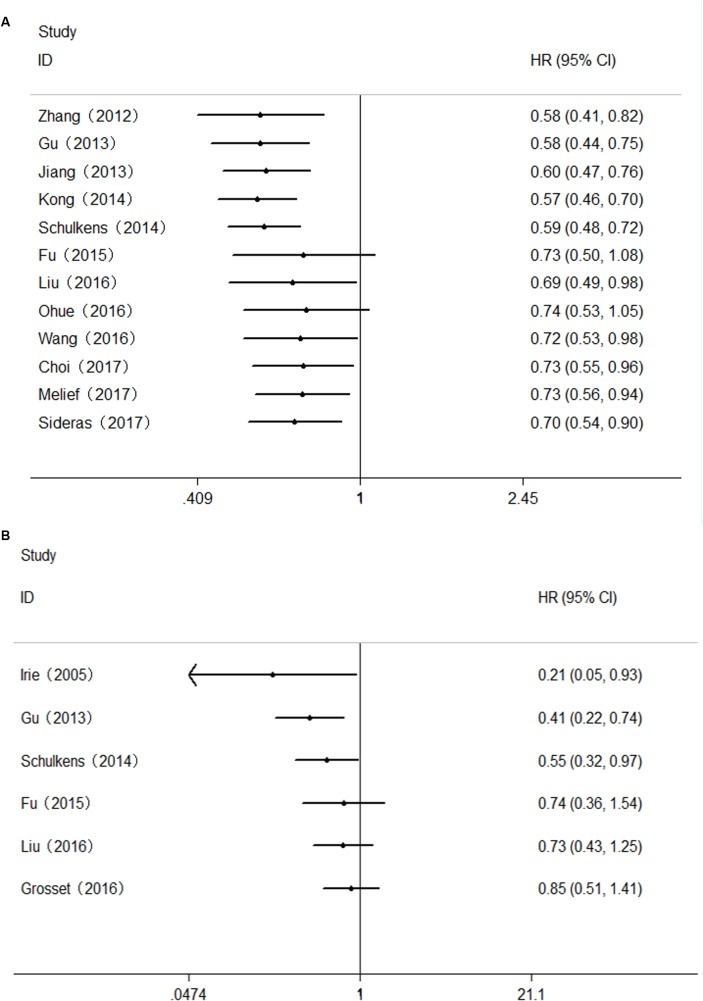
Cumulative meta-analysis of the association between Gal-9 and prognosis. **(A)** OS; **(B)** DFS/RFS.

In order to investigate the trends of the results, we also performed cumulative meta-analysis by publication year. The results indicated that the significant association between Gal-9 expression and OS became increasingly stable and that the CI narrowed since the first study performed by Zhang et al., 2012; however, these findings became inconclusive after Fu’s and Ohue’s studies were reported. As subsequent studies were reported, the data reverted to being statistically significant and became increasingly stable (**Figure 5A**).

As to DFS/RFS, the results of cumulative meta-analysis revealed that the association between Gal-9 expression and DFS/RFS became inconclusive since the Fu’s research. After two subsequent studies (Liu’s research and Grosset’s research) were reported, the CI narrowed, but the results remained inconclusive (**Figure 5B**).

### Correlation Between Gal-9 Expression and Clinicopathological Characteristics

To comprehensively analyze the role of Gal-9 expression as a biomarker in solid tumors, we investigated the association between Gal-9 expression and clinicopathological characteristics. Nine studies were included (Irie et al., 2005; Zhang et al., 2012; Gu et al., 2013; Kong et al., 2014; Fu et al., 2015; Grosset et al., 2016; Wang et al., 2016; Choi et al., 2017; Liu et al., 2017), from which 7 features were extracted for our analyses. We found that Gal-9 expression was significantly associated with the depth of invasion (TI/TII vs. TIII/TIV, OR = 2.80, 95% CI = 1.97–3.96, *P* < 0.001), histopathological stage (I/II vs. III/IV, OR = 3.00, 95% CI = 2.04–4.42, *P* < 0.001), metastasis of lymph node (Presence vs. Absence, OR = 0.47, 95% CI = 0.25–0.89, *P* = 0.020) and distal tumor metastasis (Presence vs. Absence, OR = 13.85, 95% CI = 3.50–54.76, *P* < 0.001) but not with sex, vascular invasion or tumor number (**Table 3**).

**Table 3:** Meta-analysis of the reported clinicopathologic characteristics in the included studies.

Parameters	Number of studies	Test for association			Test for heterogeneity		
			
		OR	95% CI	*p*	*I*^2^	*p*	Model
Gender (Male vs. Female)	7	0.93	[0.75–1.15]	0.509	33.8%	0.170	Fixed
Depth of invasion (TI + TII vs. TIII + TIV)	3	2.80	[1.97–3.96]	<0.001	0.0%	0.587	Fixed
Histopathological stage (I + II vs. III + IV)	3	3.00	[2.04–4.42]	<0.001	0.0%	0.918	Fixed
Lymph node metastasis (Presence vs. Absence)	6	0.47	[0.25–0.89]	0.020	78.6%	<0.001	Random
Distal tumor metastasis (Presence vs. Absence)	2	13.85	[3.50–54.76]	<0.001	0.0%	0.608	Fixed
Vascular invasion (Presence vs. Absence)	4	0.69	[0.37–1.29]	0.247	74.6%	0.008	Random
Tumor number (Single vs. Multiple)	2	1.44	[0.85–2.43]	0.180	0.0%	0.980	Fixed


**OR, odds ratio.**

### Publication Bias

**FIGURE 6:**
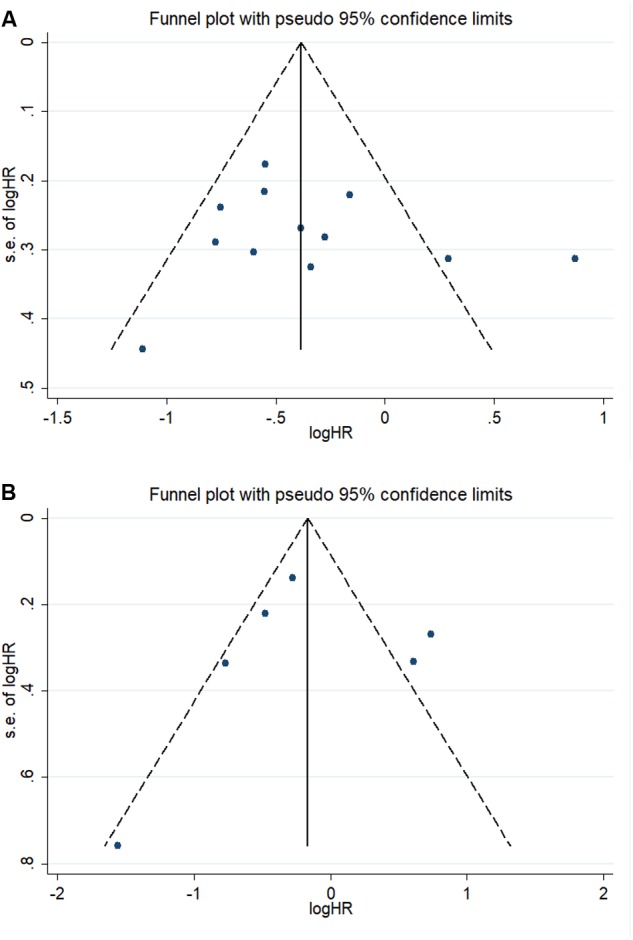
Funnel plots of the association between Gal-9 and prognosis. **(A)** OS; **(B)** DFS/RFS.

We combined funnel plots, Egger’s tests and Begg’s tests to assess whether a publication exist. Visual estimation of the funnel plots didn’t display evident asymmetry (**Figure 6**). In addition, Egger’s test (OS: *P* = 0.591; DFS/RFS: *P* = 0.980) and Begg’s test (OS: *P* = 0.537; DFS/RFS: *P* = 0.452) further confirmed no existence of publication bias.

### Sensitivity Analysis

**FIGURE 7:**
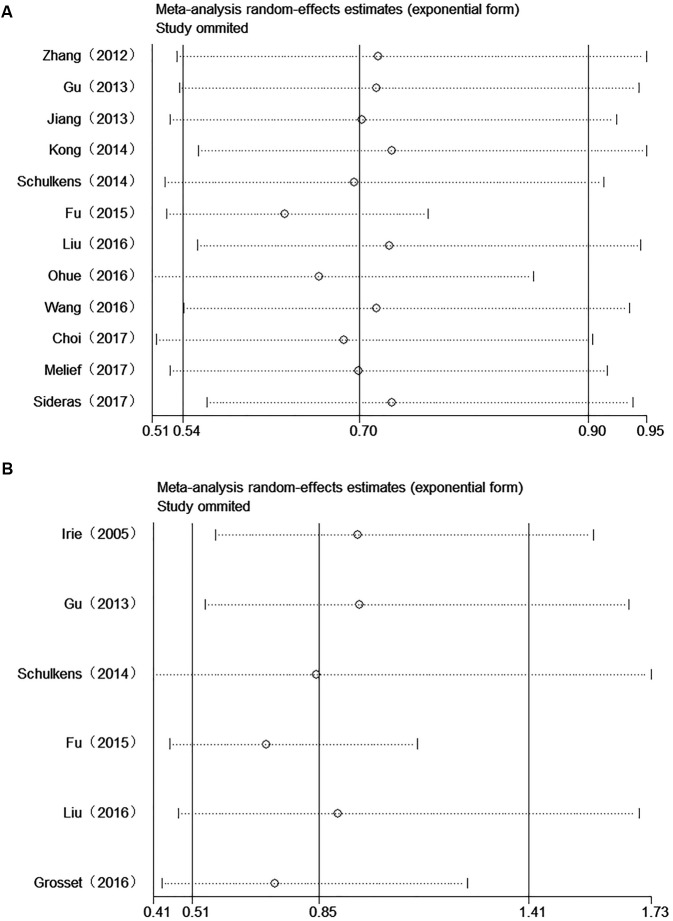
Sensitivity analysis between Gal-9 and prognosis. **(A)** Overall survival; **(B)** Disease-free survival/recurrence-free survival.

**FIGURE 8:**
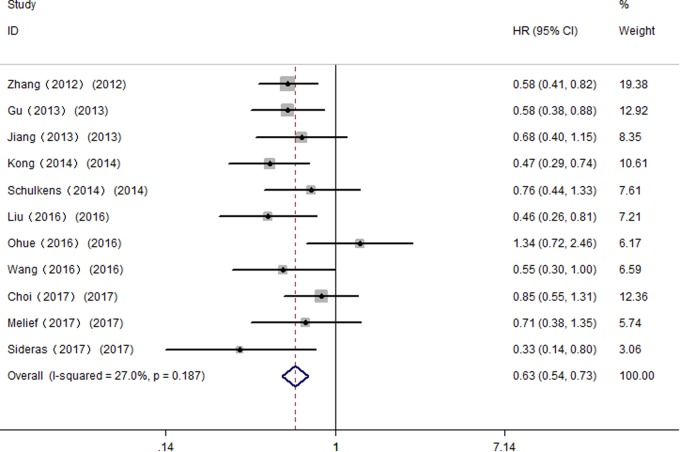
Meta-analysis of the correlation between Gal-9 and OS across homogenous studies excluding Fu’s study.

All studies were successively omitted to judge the impact of individual study on the combined results. As shown in **Figure 7**, the statistical significance of the correlation between Gal-9 and OS was not changed when any study was omitted, and the same as the inconclusive results for DFS/RFS. Furthermore, the statistical heterogeneity of the synthetic results for OS reduced remarkably when Fu’s study is removed (*I*^2^ = 27.0%, *P* = 0.187), indicating that this outlier is the source of statistical heterogeneity. After excluding Fu’s study, the remaining homogenous studies displayed a more significant correlation between high Gal-9 expression and better OS in solid tumors (HR = 0.63, 95% CI = 0.53–0.76, *P* < 0.001) (**Figure 8**), which further confirms the reliability of the results for OS.

## Discussion

Recently, multiple published studies have examined the prognostic implication of Gal-9 expression among patients with solid tumors. However, there exist controversies between the results of these studies. In the present meta-analysis, we showed that high Gal-9 expression was associated with better OS in solid cancer patients, while had no association between high Gal-9 expression and DFS/RFS. We found that in different cancer types, Gal-9 did not have a consistent effect on prognosis. High Gal-9 expression was significantly associated with better OS in HCC and colon cancer and better DFS/RFS in gastric cancer and NSCLC; however, the association remained inconclusive for other cancer types. Moreover, high Gal-9 expression was significantly correlated with a smaller depth of invasion, an earlier histopathological stage, negative lymph node metastasis and negative distal tumor metastasis. Overall, high Gal-9 expression in the tumor might improve prognosis, prevent recurrences of solid tumors, and be an emerging therapeutic target against solid tumors.

Although Gal-9 has been investigated for over 20 years, the mechanism of its role involved in improving cancer patients’ survival remains unknown. One possible explanation is its role of enhancing cell aggregation. Accumulative evidence showed that endogenous expression or exogenous administration of Gal-9 in various tumor cells could enhance cell aggregation and adhesion (Kageshita et al., 2002; Kasamatsu et al., 2005; Zhang et al., 2009). Meanwhile, increased expression of Gal-9 reduced the cell adhesion to extracellular matrix (ECM) in breast cancer cells (Irie et al., 2005), which indicated that Gal-9 expression might prevent metastasis by inhibiting the tumor cells detaching from the primary lesions and clinging to ECM. However, tumor cells also migrate as clumps with enhance cell-cell adhesion (Friedl et al., 2012). Reduced adhesion of tumor cells to ECM could at the same time allow tumor cells to more easily enter the circulation from the primary tumor location (Pandya et al., 2017). In addition, different Gal-9 variant exhibited discrepant function in regulating E-selectin expression and the cell adhesion in LoVo cells (Zhang et al., 2009). Therefore whether Gal-9 promotes or reduces metastasis in patients is still controversial, which needs further study to elucidate the underling mechanism.

Another possible explanation involves Gal-9 and its capability of inducing apoptosis. It is reported that addition of recombinant Gal-9 induced apoptosis of various malignant cells, such as hematological malignant cells (Kobayashi et al., 2010, 2015), melanoma cells (Kageshita et al., 2002; Wiersma et al., 2012) and gastrointestinal cells (Tadokoro et al., 2016, 2017; Okura et al., 2018). Further experiments performed in mouse models of leukemia (Kobayashi et al., 2010), myeloma (Kuroda et al., 2010) and hepatobiliary carcinoma (Fujita et al., 2015; Tadokoro et al., 2016) have demonstrated that treatment with recombinant Gal-9 prevents tumor progression by inducing apoptosis. However, recombinant Gal-9 is a variant that didn’t existed *in vivo*, therefore whether elevated Gal-9 expression in patients would prevent tumor metastasis in such a manner remains to be determined.

Moreover, the anti-tumor immune response of Gal-9 is also a feasible explanation. Gal-9 has been reported to strengthen antitumor immunity of CD8+ T cells cooperated with DC cells through Gal-9-Tim-3 pathway (Nagahara et al., 2008). A similar antitumor effect was observed in a murine lung cancer model, in which plasmacytoid dendritic cell-like macrophages were expanded via Gal-9 signaling (Kadowaki et al., 2012). However, Gal-9 is also implicated in tumor immune escape as described before. Thus far, it is unclear to what extend altered Gal-9 expression in patients contributes to the anti-tumor response. More studies in which galectin-9 expression is correlated with signatures of tumor infiltrating immune cells are required.

Regarding the cancer type, high expression of Gal-9 predicts favorable OS in patients with HCC and colon cancer. However, no significant correlation between Gal-9 and OS was observed for NSCLC, gastric cancer, urinary tumors and melanoma. Our finding that Gal-9 does not have the same prognostic value for different types of tumors indicates that Gal-9 might play different roles in different tumors depending on the type of cancer. For example, Gal-9 expression implies a trend toward a poor clinical outcome in urinary tumors, for both OS and DFS/RFS. This discrepant effect may be ascribed to the roles of different Gal-9 variants. Schulkens et al. (2014) reported a significant prognostic value of Gal-9 delta 5 but not of other Gal-9. Gal-9 splice variants have showed divergent roles, and thus, the role of Gal-9 may be variant dependent. Perhaps in urinary tumors, Gal-9 variants expressed in high abundance are tumor-promoting in some respects. Unfortunately, most included studies in this meta-analysis did not state specific roles for different galectin-9 variants, because of the limitation of IHC, which could not distinguish between these variants. Further studies are needed to determine the relative abundances of individual Gal-9 variants in tumors at the RNA level, by which we may link an observed association between expression and prognosis to an actual mechanism. On the other hand, most publications included in our study used IHC to detect Gal-9 and the association between Gal-9 and prognosis in these publications was significant. IHC is the primary technique used to determine protein expression in patient samples and has been widely used worldwide. Thus, our findings can be easily translated into clinical applications.

As for the subgroup analyses, high Gal-9 expression had a positive impact on prognosis in prospective studies, with concordant results for both OS and DFS/RFS. In contrast, as to retrospective studies, high Gal-9 expression showed no significant association with OS or DFS/RFS. Given that the results contained a significant heterogeneity in retrospective studies, we were cautious about the results and further performed a subgroup analysis for trial design in homogeneous population of patients with HCC. Consequently, we found that in HCC, the correlation between Gal-9 expression and OS was both significant.

We found that the prognostic value of Gal-9 for OS and DFS/RFS was not consistent. The results of a cumulative meta-analysis may provide a clue toward understanding the inconsistent results between OS and DFS/RFS. In contrast to OS, only two additional studies reporting DFS/RFS were reported since Fu’s study, and these reports narrowed the CI, but the results remained inconclusive. Therefore, we presume that as more high-quality studies reporting the correlation between Gal-9 expression and the survival of solid cancer patients become available, the data for the OS and DFS/RFS of solid tumor patients may consistently show a correlation with Gal-9 expression. In addition, as OS is well recognized as a more meaningful parameter than DFS/RFS in the survival analysis of cancer patients, we believe our study provides meaningful statistical evidence supporting the important prognostic value of Gal-9 as a favorable predictor in solid cancer patients.

In the last few decades, therapeutic strategies, such as the administration of recombinant protease-resistant Gal-9, have been implemented in preclinical studies. The results show that the administration of recombinant Gal-9 can prevent tumor progression by inducing the apoptosis of cells from various malignancies in murine models (Kobayashi et al., 2010; Kuroda et al., 2010; Fujita et al., 2015; Tadokoro et al., 2016). Our meta-analysis results further suggest that Gal-9 is not only a prognostic indicator but also an emerging therapeutic target against solid tumors. Additional studies that investigate the mechanism of the role of Gal-9’s in solid tumors are required to draw major clinical conclusions.

In the meantime, this meta-analysis is limited by several aspects. First, the results of the meta-analysis of the OS and RFS/PFS data show heterogeneity, which cannot be eliminated. We found that Fu’s study was the source of the statistical heterogeneity. Because Fu’s study investigated clear-cell renal cell carcinoma, which was distinct from other included studies. We prefer that the heterogeneity was mainly result from cancer type, probably because of the different localization of Gal-9 and different Gal-9 variants in various cancer type. In addition, there might be many other reasons for potential heterogeneity, such as the method used, sample size, follow-up time, specimen type and cutoff value, which varied among studies. However, we attempted to reduce the impact of heterogeneity through subgroup analyses. By investigating different groups of patients with homogeneous characteristics for different cancers, the results would be translatable to the clinic. Second, this meta-analysis only includes studies from Southeast Asia, North America, and New Zealand, and therefore the prognostic values of Gal-9 may not be generally applied worldwide. Finally, studies that report negative results may not have been published, which may potentially exaggerate the significance of the association between Gal-9 expression and improved prognosis. Thus, further high-quality studies regarding Gal-9 are required to resolve the abovementioned limitations.

Despite these limitations, this meta-analysis is of great significance in that it demonstrates the association between high Gal-9 expression and a better prognosis for solid tumor patients and indicates Gal-9 as a promising target against cancer therapy.

## Author Contributions

XZ designed the study, retrieved the relevant papers and data, performed the data analyses, and wrote the manuscript. LS designed the study, performed the data analyses, and participated in the writing of manuscript. DJ designed the study, assisted with the data analyses, and participated in the writing of manuscript. GX assisted with the data analyses and participated in the writing of manuscript. JmZ designed the study and retrieved the relevant papers and data. LL and JjZ designed the study and retrieved the relevant papers and data. ZY retrieved the relevant papers and data and assisted with the data analyses. HL designed the study, assisted with the writing of manuscript, and supervised the study. The final approval of all authors was reached.

## Conflict of Interest Statement

The authors declare that the research was conducted in the absence of any commercial or financial relationships that could be construed as a potential conflict of interest.

## Footnotes

1 http://digitizer.sourceforge.net/
